# Primary Cutaneous Diffuse Large B-Cell Lymphoma of the Upper Limb: Double Hit/Double Expressor with CNS Involvement: From Hospice to Remission

**DOI:** 10.1155/2019/3953470

**Published:** 2019-02-07

**Authors:** Brenda S. Castillo, Maria L. Rodriguez, Ju-Hsien John Chao, Behyar Zoghi

**Affiliations:** ^1^Texas Tech University Health Sciences Center Paul L. Foster School of Medicine, El Paso, Texas, USA; ^2^Brown University, Providence, RI, USA; ^3^Methodist Hospital, Texas Transplant Institute, San Antonio, Texas, USA

## Abstract

We report a patient with diffuse large B-cell lymphoma of skin, nongerminal center type double hit double expressor, with an initial presentation as a left forearm mass. The patient underwent chemotherapy after initial diagnosis. After chemotherapy regimen, she developed a second mass, followed by CNS involvement with neurological defects. At this time, a three line of chemotherapy was used with minimal effects. The patient was deemed terminal and was recommended hospice care. The patient decided to continue with skin and crainospinal radiotherapy and intrathecal chemotherapy; she achieved complete remission. After achieving complete remission, the patient underwent an autologous stem cell transplant with minimal transplant-related toxicity.

## 1. Introduction

Diffuse large B-cell lymphoma (DLBCL) is the most common type of non-Hodgkin lymphoma (NHL), accounting for 30–58% of all cases [1]. High-grade B-cell lymphoma (HGBL) is a new World Health Organization classification of high-risk DLBCL, characterized by double hit (DH) or triple hit MYC and BCL2 and/or BCL6 rearrangement [2]. Different types of chemotherapy regimens such as R-CHOP (rituximab, cyclophosphamide, doxorubicin, vincristine, and prednisone) and DA-R-EPOCH (dose adjusted rituximab, etoposide, prednisone, cyclophosphamide, and doxorubicin) have been used with less response [3]. The nongerminal center subtype of HGBL-DH occurs in 1.7% of all DLBCL patients, and it presents with MYC/BCL6 rearrangements more often than MYC/BCL2 rearrangements [4]. There are no significant prognostic differences between MYC/BCL2 and MYC/MCL6 outcomes, and there is no established difference or similarity between each subtype of HGBL-DH [5]. Given the high risk of relapse of disease, maintenance therapy using lenalidomide or venetoclax was attempted and has had positive results, but there is no recommended primary therapy as of yet [6].

We present a case with characteristics of DLBCL with primary skin involvement. There are five types of primary cutaneous B-cell lymphoma: marginal zone primary cutaneous B-cell lymphoma, centrofollicular primary cutaneous lymphoma, diffuse large B-cell primary cutaneous lymphoma-leg type, large B-cell primary cutaneous lymphoma (NOS), and intravascular large B-cell primary cutaneous lymphoma [7]. The case we present behaves like cutaneous DLBCL leg type, which is the most aggressive subtype, with MYC and BCL2/BCL6 expression. DLBCL leg type presents with red to bluish nodules or tumors on one or both lower legs. Only about 10% to 15% of these patients are noted to develop lesions outside of the lower extremities, as was the case with our patient, who presented with a left arm nodule [4]. DLBCL leg type tumors are more aggressive with worse outcomes, since they frequently disseminate to lymph nodes. Given the relapse and progression of the disease, which was initially noted in the skin and immunohistochemistry analysis, our case behaved as DLBCL leg type. Lymph node involvement was secondary since the only lymph nodes involved are the axillary lymph nodes, the most proximal to the cutaneous involvement. Chemotherapy by itself had minimal effect in controlling the disease. Only after the addition of radiation therapy was complete remission achieved. The patient subsequently received autologous stem cell transplant as consolidation therapy.

## 2. Case Presentation

**Figure 1:**
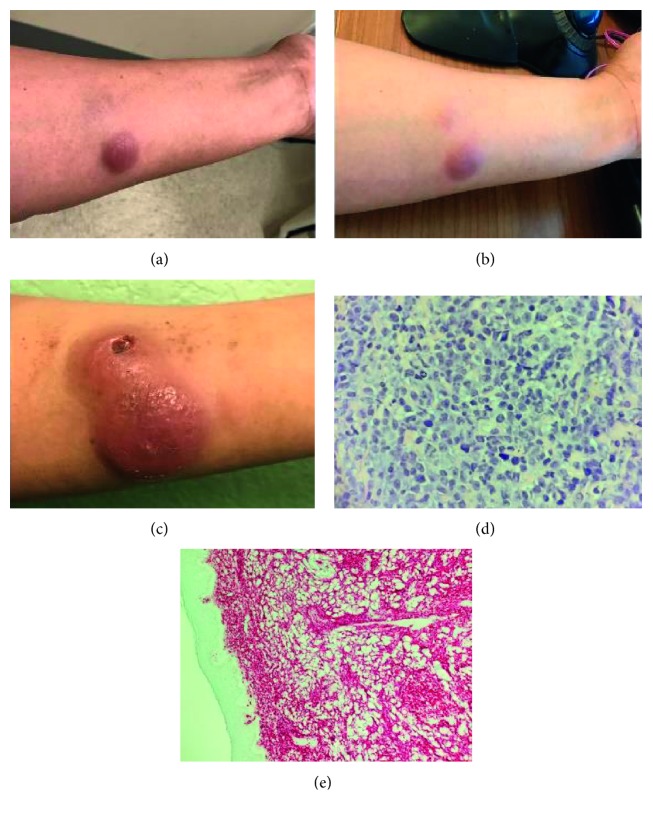
Left forearm masses. (a) Initial mass at time of antibiotic treatment on 03/03/17. (b) Appearance of the second lesion on 03/08/17. (c) Lesion appearance on 03/30/17. Skin biopsy representing cutaneous (d) DLBCL and (e) CD20 staining.

In 2016, a 39-year-old healthy woman began having night sweats, and within the next month, she discovered an erythematous, round skin lesion in her left, middle forearm. She presented to her primary care provider with a progressively growing erythematous nodule and was treated with antibiotics (Figure 1(a)). While the initial lesion continued to grow, a second lesion appeared next to the first (Figure 1(b)). The patient was evaluated by a dermatologist, and two biopsies were obtained. The patient was diagnosed with DLBCL non-GC subtype (Figures 1(c)–1(e)). CT showed left axillary lymphadenopathy with lymphoma confined to the left forearm. Bone marrow biopsy showed no lymphoma. PET scan showed lymphoma in the left forearm and left axilla.

**Figure 2:**
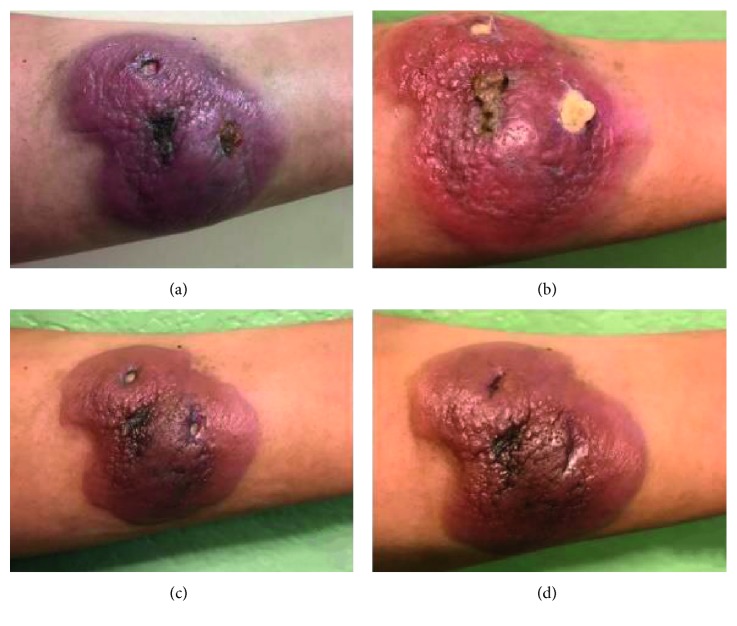
Left forearm tumor. (a) Before receiving first R-CHOP regimen on 04/19/17. (b) After the first R-CHOP cycle on 04/22/17. (c) After the second R-CHOP cycle on 05/11/17. (d) Left forearm at the end of the third cycle on 07/14/17 R-CHOP cycle on 06/01/17.

**Figure 3:**
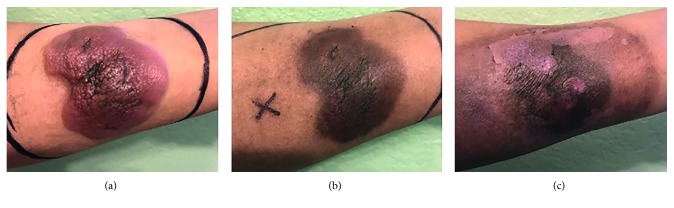
Left forearm during radiotherapy. (a) Left forearm at the beginning of radiotherapy on 06/05/17. (b) Left forearm at the end of radiotherapy on 06/25/17. (c) Left forearm at the end of the fifth R-CHOP cycle on 07/14/17.

The patient began R-CHOP regimen on April 2017. Following three cycles of R-CHOP, the tumor continued to grow (Figure 2). In addition to the R-CHOP regimen, radiotherapy to the left forearm begun in May 2017. After the fifth R-CHOP cycle and radiotherapy completion, the tumor began to shrink (Figure 3).

**Figure 4:**
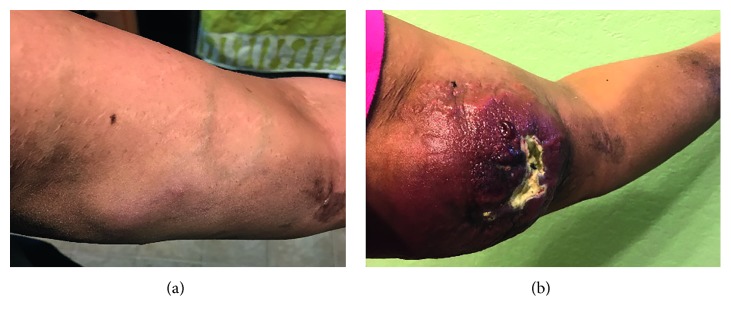
(a) Left upper arm mass on 08/11/17. (b) Left upper arm mass on 11/05/17.

Unfortunately, one month later, the patient noted a nodule on her left upper arm (Figure 4(a)). Two more cycles of R-CHOP were administered. In the next month, restaging PET/CT showed an increase in nodularity of the left forearm, indicating progressive lymphoma. Fine-needle aspiration (FNA) of the left forearm lesion was positive for DLBCL non-GC subtype. The R-CHOP regimen was stopped, and the patient received one cycle of E-SHAP (etoposide, methylprednisolone, and cytarabine). Skin masses and nodules in her left upper arm continued to grow fast within 3 months (Figure 4(b)). The regimen was changed to rituximab, gemcitabine, and oxaliplatin with no response. The patient was evaluated by the bone marrow transplant team at the Methodist hospital and was referred to MD Anderson Cancer Center (MDACC) to participate in a chimeric antigenic receptor (CAR) T-cell clinical trial.

**Figure 5:**
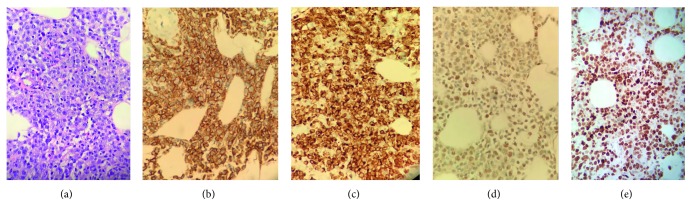
(a) H&E staining of diffuse large B-cell lymphoma; (b) CD20 immunostaining; (c) BCL2 immunostaining; (d) BCL6 immunostaining; (e) MYC immunostaining.

Lymph node (left axilla) needle biopsy at MDACC was diagnostic for DLBCL non-GC subtype immunophenotype. The neoplasm showed a diffuse pattern composed predominantly of large cells with immunoblastic cytological features. Immunohistochemical studies were reviewed, and the neoplastic cells were positive for CD19, CD20, PAX-5, BCL2, BCL6, and MUM-1/IRF4. Ki-67 showed a proliferation rate of 70–80%, negative for Epstein–Barr virus-encoded small RNA (EBER). Immunohistochemistry using c-MYC antibody showed 70–80% of neoplastic cells weakly to moderately positive for MYC. Combined with the BCL2 result, the neoplasm was a double expressor immunophenotype (Figure 5). MYC FISH analysis showed a positive result for MYC gene rearrangement. IGH BCL2 FISH was negative for IGH/BCL-2 gene rearrangement. BCL6 FISH was positive for BCL6 gene rearrangement with 70% of the interphase indicating BCL6 rearrangement.

**Figure 6:**
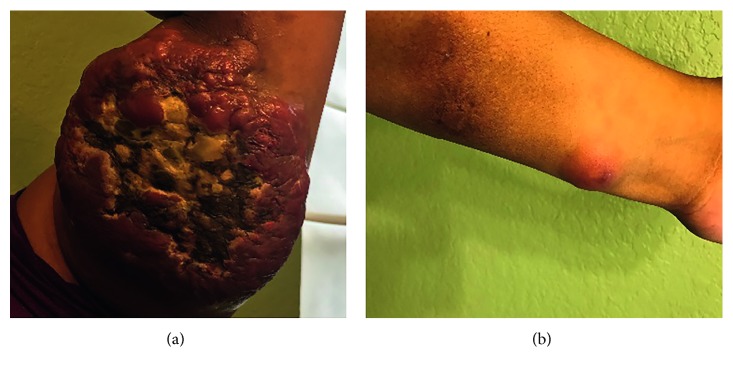
(a) Left upper arm mass 12/04/17. (b) Third nodule appearance on left forearm 12/18/17.

Although the patient's stem cells were collected in November 2017 and only CD4 cells were amplified, she did not receive the infusion. She developed Bell's palsy of the right side of her face that was attributed to a viral illness for which she received treatment. She also developed double vision. Lumbar puncture showed atypical lymphoid cells, consistent with involvement by patient's lymphoma. CSF analysis by flow cytometry was positive for large number of aberrant B cells with partial CD10 expression. Aberrant cell phenotype was positive for CD10, CD19, CD20, and CD22. At the beginning of December, the left upper arm mass had continued to grow (Figure 6(a)). She received high-dose methotrexate. Days later, a third nodule was present in the patient's left forearm (Figure 6(b)). At the end of the month, she was admitted to MDACC ER, was unable to walk, and had generalized weakness.

**Figure 7:**
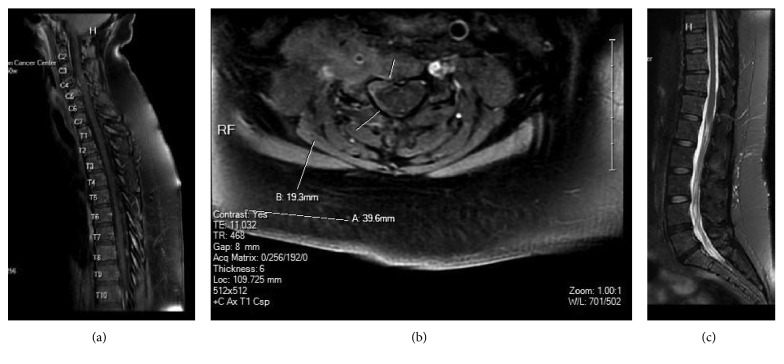
(A and B) MRI cervical and thoracic showed C5 metastasis that extended into the right C4-C5 and C5-C6 foramina (C) with leptomeningeal metastasis.

Brain MRI showed no acute intracranial abnormality or intracranial metastasis. Lumbar puncture CSF showed large malignant cells, consistent with involvement by the patient's known DLBCL. Cervical, thoracic, and lumbar MRI showed C5 metastasis that extended into the right C4-C5 and C5-C6 foramina. No spinal cord compression was observed. Days later, lumbar puncture CSF showed large malignant lymphoid cells, morphologically consistent with involvement by patient's known DLBCL. CSF flow cytometry was positive for lymphoma with aberrant cell phenotype positive for CD19, CD20, CD22, CD38, CD43, CD79b, and CD200. Cervical, thoracic, and lumbar MRI showed an enhancing mass involving the right C5 vertebral body with extension into the right C4-C5 foramina (Figures 7(a) and 7(b)) and epidural extension of tumor along the right aspect of the spinal canal touching the right aspect of the cord and leptomeningeal metastasis (Figure 7(c)). She received Ferreri regimen (high dose of methotrexate with high-dose cytarabine) followed by intrathecal methotrexate. Lumbar puncture CSF showed rare atypical cells, suspicious for involvement by patient's known large B-cell lymphoma. The patient was told that was terminal and advised to seek comfort measures.

She decided to continue treatment and radiotherapy to the left arm, 3000 cGy for 10 cycles. After beginning left arm radiotherapy, she received craniospinal radiation 2000 cGy for 4 cycles. She began to walk again, and at the time, her left forearm tumor had subsided. Lumbar puncture CSF in the middle of January showed no malignant cells.

**Figure 8:**
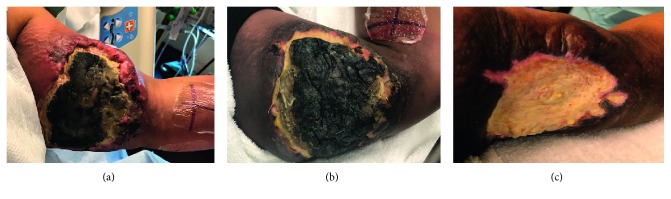
Left upper arm during radiotherapy. (a) Left upper arm at the beginning of radiotherapy on 01/07/18. (b) Left upper arm after completing radiotherapy on 01/11/18. (c) Left upper arm after excision of mass on 02/27/18.

**Figure 9:**
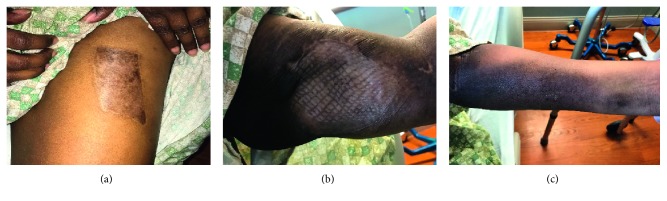
(a) Region of left thigh where skin graft was obtained. (b) Left upper arm with skin graft. (c) Left forearm.

In February 2018, the patient had a left upper arm mass infection, treated with IV antibiotics, and the left arm tumor mass was excised (Figure 8). She received a skin graft from her left thigh (Figure 9). Biopsy showed no evidence of lymphoma.

A month later, CSF showed revealed no lymphoma. PET/CT, skull base to midthigh, showed soft nodular recurrence in the patient's left arm, as well as asymmetric uptake in the patient's right shoulder musculature. She was reevaluated by MDACC in order to continue her involvement in CAR T-cell clinical trial. The patient had regained the ability to walk, and talk without limitation, but had a residual left facial droop. Tumor status was as assessed as complete remission by PET; external CSF reports are negative for lymphoma. After evaluation, it was confirmed that she was in remission. In May 2018, CSF revealed no lymphoma. Bone marrow biopsy showed no blasts or lymphoma, and it showed hypocellular 35–40% marrow with active progressive trilineage hematopoiesis. Her left forearm and left upper arm skin had healed (Figure 8). She received consolidation therapy with autologous bone marrow transplant, with conditioning regimen of rituximab, thiotepa, and carmustine. The patient was engrafted by day 12 and had a readmission shortly for *C. diff* diarrhea which was treated appropriately. The patient was subsequently discharged and is followed as an outpatient.

## 3. Discussion

The standard therapy for patients with DLBCL non-GC subtype is R-CHOP. Our patient presented with DLBCL non-GC subtype on the left forearm and was initially treated with R-CHOP. Her condition deteriorated, and treatment regimen was switched to E-SHAP, then to rituximab, gemcitabine, oxaliplatin, and high-dose methotrexate, showing no resolution. Her disease progressed to involve the CNS, leaving her unable to walk. Upon treatment with radiotherapy, she went into remission. Immunohistochemistry and cytogenetic analysis of the patient revealed expression of MYC and BCL2, along with MYC and BCL6 gene rearrangements. The use of radiotherapy caused left forearm, left upper arm, and CNS lymphoma eradication. Immunohistochemistry and cytogenetic analysis should be considered when deciding on treatment modalities, given that expression of MYC and BCL2 (double hit lymphoma) and MYC and BCL6 gene rearrangement, make disease progression not only more aggressive in course, and higher risk to involve the CNS, but it is also associated with poor outcomes using the R-CHOP regimen [3]. Radiotherapy should be considered as a first-line treatment combined with chemotherapy. Other alternatives include intensifying current therapies using dose-adjusted DA-R-EPOCH, which has been associated with better outcome for double hit DLBCL that can be substituted for R-CHOP [5]. The use of BCL2 inhibitors, such as venetoclax, could serve as an agent for maintenance therapy. A potent, selective, and orally bioavailable agent, venetoclax, has been used in clinical trials to evaluate its efficacy in relapsed or refractory cases of NHL [6]. In addition, lenalidomide has been shown to induce responses in both nongerminal center and germinal center DLBCL, showing superior responses in nongerminal center DLBCL [4]. Our patient experienced further tumor reduction and CNS involvement with the use of radiotherapy. She regained the ability to walk, attained remission, and received autologous stem cell transplant. Given very aggressive mature of this type of lymphoma and high risk of relapse, maintenance therapy around day 100 posttransplant is suggested after patient full engraftment. This case shows that radiation therapy should be incorporated early on as it showed a significant effect in achieving remission in our patient. This case should ignite a new standard of care for this type of lymphoma.
